# Autophagy Contributes to the Maintenance of Genomic Integrity by Reducing Oxidative Stress

**DOI:** 10.1155/2020/2015920

**Published:** 2020-08-25

**Authors:** Wenqing Bu, Xiaohe Hao, Tingting Yang, Jing Wang, Qiao Liu, Xiyu Zhang, Xi Li, Yaoqin Gong, Changshun Shao

**Affiliations:** ^1^State Key Laboratory of Radiation Medicine and Protection, Institutes for Translational Medicine, Soochow University, Suzhou, Jiangsu 215123, China; ^2^MOE Key Laboratory of Experimental Teratology, Department of Genetics, Shandong University School of Basic Medical Sciences, Jinan, Shandong 250012, China

## Abstract

Autophagy has been well documented to play an important role in maintaining genomic stability. However, in addition to directly engulfing and digesting the damaged organelles and chromatin fragments, autophagy can affect many cellular processes including DNA damage response, regulation of redox homeostasis, and cell division; it remains to be determined to what extent each of those processes contributes to the maintenance of genomic stability. We here examined the role of autophagy-dependent redox regulation in the maintenance of genomic stability in two cancer cell lines (HT1080 and U2OS) and mesenchymal stem cells (MSCs) using micronuclei MN, also referred to as cytoplasmic chromatin fragments, as a marker. Our results showed that the spontaneous and genotoxic stress-induced frequencies of MN in cancer cells were significantly reduced by autophagy activators rapamycin and Torin1, and the reduction in MN was accompanied by a reduction in reactive oxygen species (ROS). Increased micronucleation in senescent MSCs, in which autophagic flux is blocked, was also attenuated by rapamycin, together with a reduction in ROS. Inhibition of autophagy by chloroquine (CQ) or ATG5 depletion, on the other hand, resulted in an increased frequency of MN, though a ROS elevation in response to autophagy inhibition was only observed in MSCs. Importantly, the induction of MN by autophagy inhibition in MSCs could be abrogated by antioxidant N-acetylcysteine (NAC). In contrast to the reported impairment of CHK1 activation in Atg7-deficient mouse embryonic fibroblasts, we found that the level of phosphorylated CHK1 was increased by CQ or ATG5 depletion but decreased by rapamycin or Torin1, suggesting that the increased genomic instability by defective autophagy is not caused by insufficient activation of CHK1-homologous recombination cascade. Together, our findings suggest that redox homeostasis regulated by autophagy contributes substantially to the maintenance of genomic stability in certain contexts.

## 1. Introduction

Macroautophagy, hereafter known as autophagy, is a lysosomal-dependent degradation pathway that involves in degrading and recycling redundant or damaged substances to protect cells from various types of stress [1]. Autophagy occurs in the cytoplasm and yet affects genetic material in the nucleus [2]. Impaired autophagy may lead to damaged or disrupted genome and result in diseases such as neurodegenerative diseases and cancer [3, 4]. Therefore, it is particularly crucial to understand how autophagy contributes to the maintenance of genomic stability.

Micronuclei (MN), also known as cytoplasmic chromatin fragments (CCFs), serve as a sensitive indicator of genomic instability as well as the extent of DNA damage [5]. In addition, it has been reported that MN can activate cGAS-STING signaling pathway and trigger a series of important cellular processes such as innate immune response, cell senescence, and disturbance of DNA damage repair [6–8]. Therefore, it is particularly important to explore how the kinetics of micronuclei is regulated. Rello-Varona et al. reported the presence of “autophagic micronuclei,” micronuclei that exhibit autophagic marker GFP-LC3 [9]. The micronucleation is usually exacerbated in cells with defective autophagy [10, 11]. However, these autophagic micronuclei account for only a very small proportion of the MN [9]. In addition to directly disposing and recycling the damaged organelles, proteins, and chromatin fragments, autophagy is also known to affect other cellular processes that may affect genome stability. The accumulation of DNA lesions in autophagy-deficient cells could be driven by a defect in DNA repair capability [12]. Autophagy is reported to contribute to genome stability through modulation of cell division under conditions that inhibit cell growth [13] and by degrading retrotransposon RNA [14]. Autophagy can also affect the regulation of redox homeostasis [15–17]. Therefore, it remains to be determined to what extent each of those processes regulated by autophagy contributes to the maintenance of genomic stability.

Reactive oxygen species (ROS) are critical for sustaining many cellular processes, and their generation and scavenging need to be tightly regulated to maintain the redox homeostasis [18]. Once their production overwhelms the antioxidant system, oxidative stress ensues and causes various types of DNA damage including base damage, single-strand breaks, and double-strand breaks. Oxidative stress preferentially induces the formation of MN-*γ*-H2AX(+), a subtype of MN that shows uniform phosphorylation of H2AX at serine 139 [19]. Therefore, oxidative stress is one of the most significant causes of genomic instability [17]. Interestingly, redox regulation and autophagy are interrelated. On the one hand, ROS have been reported to mediate the induction of autophagy caused by nutrient deprivation [20]. Impaired autophagy, on the other hand, can lead to increased oxidative stress. Dysfunctional autophagy in cancer cells results in mitochondrial damage and ROS accumulation [21]. Hematopoietic stem cells in Vav-Atg7^−/−^ mice accumulate mitochondrial superoxide [22]. However, autophagy is involved in the degradation of catalase, resulting in the accumulation of ROS [23]. Therefore, the regulation of cellular ROS level by autophagy may vary with contexts; the roles of redox homeostasis in the maintenance of genomic stability by autophagy are not clear.

We here explored the role of autophagy in genomic stability and tested whether it is mediated by redox regulation by using micronuclei as a marker. We showed that spontaneous and genotoxic stress-induced micronucleation can be reduced by autophagy activators, which is accompanied by a decrease in ROS. Elevated micronuclei and ROS level in senescent cells, in which autophagic flux is blocked, were also reduced by rapamycin. At the same time, we also observed an increase in ROS and MN in MSCs after autophagy is inhibited. Importantly, antioxidant effectively rescued micronuclei elevation caused by autophagy inhibition. Thus, the elevation of ROS caused by impaired autophagy can contribute to genomic instability in certain contexts.

## 2. Materials and Methods

### 2.1. Cells and Cell Culture

Human cancer cell lines HT1080 and U2OS were obtained from the Cell Bank of Chinese Academy of Sciences (Shanghai, China). Human umbilical cord-derived mesenchymal stem cells (hUC-MSCs) were as described [24]. HT1080 cells were cultured in the minimum Eagle's medium (Gibco, USA). U2OS cells were cultured in the Dulbecco's modified Eagle's medium (Gibco, USA). MSCs were cultured in the Minimum Essential Medium-*α* (*α*-MEM, Gibco, USA); all medium was supplemented with 10% FBS, 100 U/mL penicillin, and 100 *μ*g/mL streptomycin. *α*-MEM was also supplemented with EGF, bFGF, VEGF, and PDGF (each at 2 ng/mL, Proteintech, USA). Cells were maintained in a humidified 5% CO_2_ atmosphere at 37°C.

### 2.2. Cell Treatments

Rapamycin and Torin1 were purchased from Beyotime Institute of Biotechnology (China). Chloroquine (CQ) and N-acetylcysteine (NAC) were purchased from Selleck Chemicals (USA). Hydroxyurea (HU) was from Sigma-Aldrich (USA). X-ray apparatus (X-RAD 225 OptiMAX) was from Precision X-ray (PXi).

### 2.3. Immunofluorescence Analysis

The cells grown on coverslips in 6-well plates were washed in PBS twice and fixed in Immunol Staining Fix Solution (Beyotime, China, P0098) for 15 min. After being washed three times in PBS, cells were permeabilized for 15 min in 0.2% Triton X-100 PBS (PBST) and then blocked in 10% normal goat serum overnight at 4°C. After three times of washing in PBST, the coverslips were incubated with primary antibody overnight at 4°C, then washed in PBST, and incubated with TRITC-conjugated secondary antibody (1 : 200, Jackson ImmunoResearch Laboratories, West Grove, PA) for 1 h at room temperature in the dark. The coverslips were washed in PBST three times and counterstained with mounting medium containing DAPI (Abcam, ab104139) and then were mounted on slides for examination under a fluorescence microscope. Antibody for immunofluorescence detection of phospho-H2AX was purchased from Millipore Corp (1 : 500, Millipore Corp, USA, 2854975); Lamin B1 was from Proteintech (1 : 200, Proteintech, 12987-1-AP).

### 2.4. Scoring of MN

The coverslips were examined under an Olympus DP71 fluorescence microscope. Fields were randomly selected and focused to reveal DAPI staining under the 40x objective. Nuclei and MN were identified by staining by DAPI, and at least 1000 cells in each sample were counted. The MN-*γ*-H2AX (+) were then identified and scored by the presence of intensive and uniform *γ*-H2AX staining. Cells with three or more MN were not included to avoid bias caused by catastrophic cellular events [25].

### 2.5. Western Blot Analysis and Antibodies

Cells were harvested and lysed with the lysis buffer (Beyotime, China, P0013) plus protease inhibitors on ice for 30 min. The lysates were centrifuged at 12,000 rpm for 15 min to remove all cellular debris. Protein concentrations were determined by BCA assay kit (Beyotime, China, P0012). Equal amounts of samples were run on SDS-PAGE gels and transferred to PVDF membrane (Millipore, Billerica, MA, USA). The membrane was blocked with 10% skim milk for 1 h at room temperature and then incubated with primary antibodies for overnight. Antibodies against *γ*-H2AX (Ser139, 1 : 1000, 9718T), LC3A/B (1 : 1000, 12741S), and p-CHK1 (Ser345, 1 : 1000, 2348S) were purchased from Cell Signaling Technology (USA). Anti-ATG5 (1 : 1000, 10181-2-AP), anti-GAPDH (1 : 5000, 60004-1-Ig), and anti-*α*-Tubulin (1 : 5000, 66031-1-Ig) were from Proteintech (USA). Anti-p62-SQSTM1 (1 : 1000, ab207305) was from Abcam (UK). Anti-CHK1 (1 : 200, sc-8408) was from Santa Cruz Biotechnology (USA). The proteins of interest were visualized after incubation of membranes with the appropriate horseradish peroxidase secondary antibody by ECL kit (Thermo, USA).

### 2.6. Measurement of Cellular ROS by Flow Cytometry

Cellular ROS level was measured by Reactive Oxygen Species Assay Kit (Beyotime, China, S0033). After being subjected to any of the treatments, the cells were harvested and washed and then stained with 10 *μ*M DCFH-DA probe for 20 min at 37°C in a humidified atmosphere at 5% CO_2_. After being washed twice in PBS, cells were harvested and fluorescence intensity of DCF was examined using flow cytometry (FACSCanto II, BD, USA).

### 2.7. RNA Interference

The small interfering RNAs (siRNAs) were purchased from GenePharma. Cells were transfected with siRNAs (50 nM) using Lipofectamine 2000 (Invitrogen, USA) following the manufacturer's guide. Interference efficiency was determined 48 h after transfection.

The siRNAs sequences are as follows:

*ATG5*: sense strand 5′-GGAUCAACUAUUUGCCUGAdTdT-3′; antisense strand 5′-UCAGGCAAAUAGUUGAUCCdTdT-3′

*Scramble control*: sense strand 5′-UUCUCCCGAACGUGUCACGUTTdTdT-3′; antisense strand 5′-ACGUGACACGUUCGGAGAATTdTdT-3′

### 2.8. SA-*β*-Gal Staining

The proportion of senescent cells was evaluated using the SA-*β*-gal Staining Kit (Beyotime, China, C0602) according to the manufacturer's protocols. The cells were incubated overnight at 37°C in a humidified atmosphere and then washed with PBS and scored under a microscope. At least 500 cells were counted for each sample.

### 2.9. Statistical Analysis

Data were shown as mean ± SD. The statistical significance was determined by Student's *t*-test. *P* < 0.05 was considered statistically significant. ∗ indicates *P* < 0.05; ∗∗ indicates *P* < 0.01; ∗∗∗ indicates *P* < 0.001; and ∗∗∗∗ indicates *P* < 0.0001.

## 3. Results

### 3.1. Genotoxicants Induce Micronucleation

**Figure 1:**
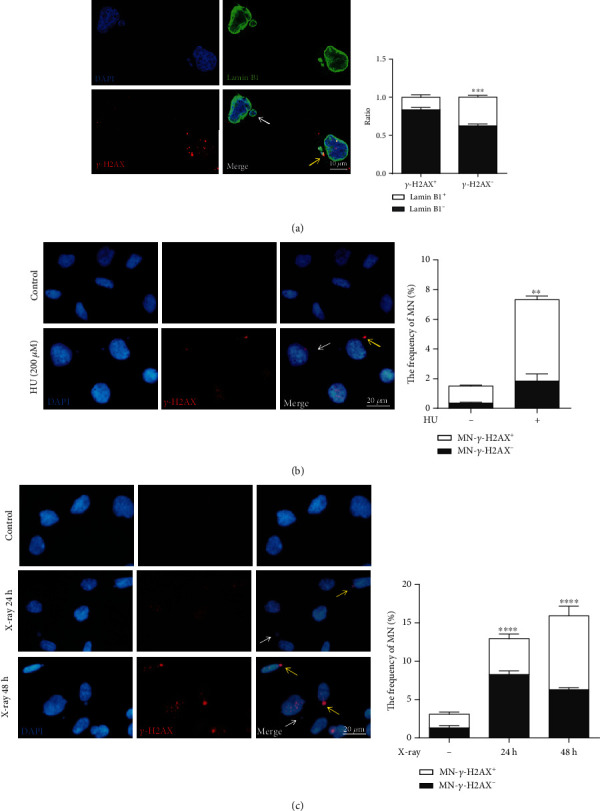
Micronuclei can be induced by genotoxicants. (a) Comparison of micronuclei membrane integrity between MN-*γ*-H2AX (+) and MN-*γ*-H2AX (-) in HT1080 cells. Representative images showing Lamin B1 (green) on the nuclear envelope, *γ*-H2AX (red) marking DNA double-strand break foci, and DAPI staining of DNA (blue). At least 200 MN of each type were counted. (b) The frequencies of MN measured by immunofluorescence in HT1080 cells treated with 200 *μ*M HU for 24 h and washed out for 48 h. The left pictures show representative images. (c) The frequencies of MN in HT1080 cells at 24 h and 48 h after 10 Gy X-ray. The left pictures show representative images. Yellow arrows indicate MN-*γ*-H2AX (+); white arrows indicate MN-*γ*-H2AX (-). Each experiment was repeated three times. ^∗∗^*P* < 0.01, ^∗∗∗^*P* < 0.001, and ^∗∗∗∗^*P* < 0.0001. MN: micronuclei; HU: hydroxyurea.

MN-*γ*-H2AX (+) are usually associated with cGAS due to ruptured nuclear envelope [6]. We first examined the envelope integrity in MN-*γ*-H2AX (+) and MN-*γ*-H2AX (-). Nuclear envelope protein Lamin B1 and *γ*-H2AX were examined by immunofluorescence. As shown in Figure 1(a), compared with MN-*γ*-H2AX (-), the nuclear envelopes of MN-*γ*-H2AX (+) tend to be less intact. We treated HT1080 cells with 200 *μ*M HU, an inducer of replication stress, for 24 h and examined the frequencies of the MN-*γ*-H2AX (+) at 48 h after HU was washed out. As shown in Figure 1(b), HU treatment led to a significant increase in frequency of MN. Next, we treated cells with X-ray (10 Gy) and evaluated the frequencies of MN at 24 h and 48 h, respectively. The MN frequencies at both time points were elevated significantly, and MN-*γ*-H2AX (+) became predominant at 48 h (Figure 1(c)). These results confirm our previous findings that MN can be effectively induced by genotoxicants [25].

### 3.2. Augmentation of Autophagy Reduces Spontaneous and Genotoxic Stress-Induced Micronuclei in Cancer Cells

**Figure 2:**
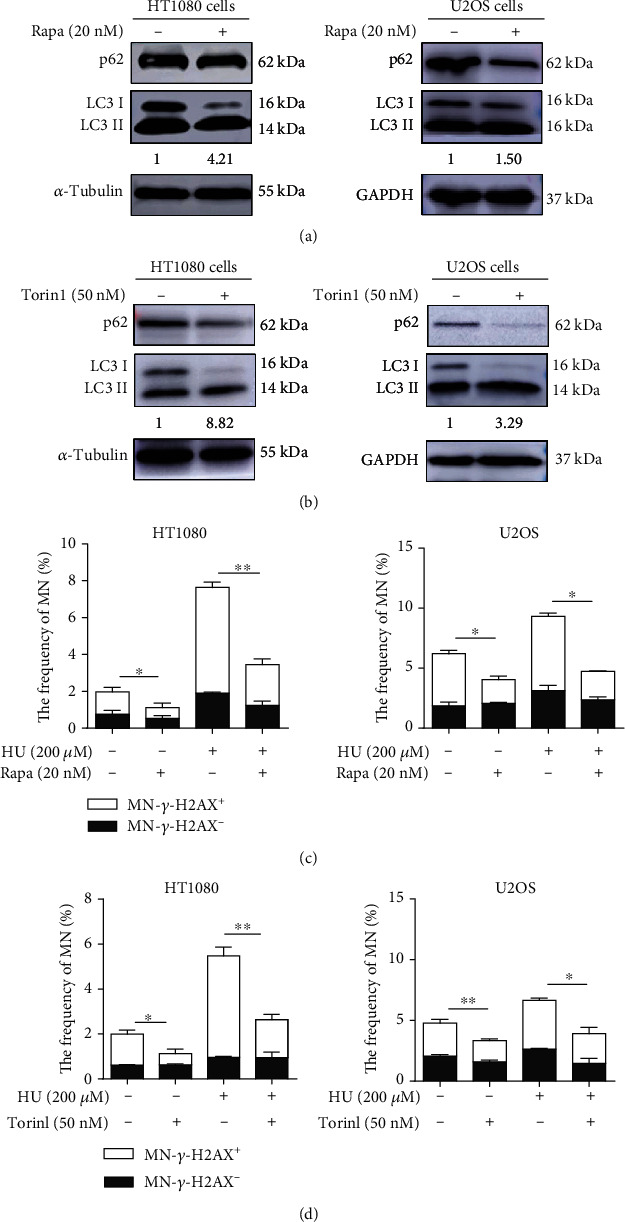
Autophagy reduces spontaneous and replication stress-induced micronuclei. (a, b) Rapa or Torin1 treatment effectively activates autophagy. HT1080 cells and U2OS cells were treated with autophagy activator Rapa (20 nM) or Torin1 (50 nM) for 24 h, and cells were harvested for measurement of autophagy efficiency (LC3 and p62) by Western blot analysis (the numbers below indicate the ratios of LC3-II/LC3-I). (c, d) Autophagy activators reduce spontaneous and replication stress-induced micronuclei. HT1080 cells and U2OS cells were treated with 20 nM Rapa and 50 nM Torin1 or cotreated with 200 *μ*M HU for 24 h. The frequencies of MN were tested at 48 h after drugs were washed out. Each experiment was repeated three times. ^∗^*P* < 0.05 and ^∗∗^*P* < 0.01. Rapa: rapamycin.

We treated cells with rapamycin (Rapa), a classic autophagy activator [26], and confirmed the induction of autophagy by Western blot analysis (Figure 2(a)). Torin1, another activator of autophagy, was also effective in inducing autophagy (Figure 2(b)). We then tested the effect of augmented autophagy on the frequency of spontaneous and HU-induced MN. HT1080 and U2OS cells were treated with 20 nM rapamycin and/or 200 *μ*M HU for 24 h. The frequencies of MN were scored at 48 h after drugs were washed out. As shown in Figure 2(c), the frequencies of spontaneous and replication stress-induced MN of HT1080 cells and U2OS cells were greatly reduced by rapamycin. Torin1 similarly reduced the frequencies of MN in HT1080 and U2OS cells (Figure 2(d)). Rapamycin also attenuated the X-ray-induced micronuclei (Figures S1(a) and S1(b)).

### 3.3. Autophagy Reduces ROS in Cancer Cells

**Figure 3:**
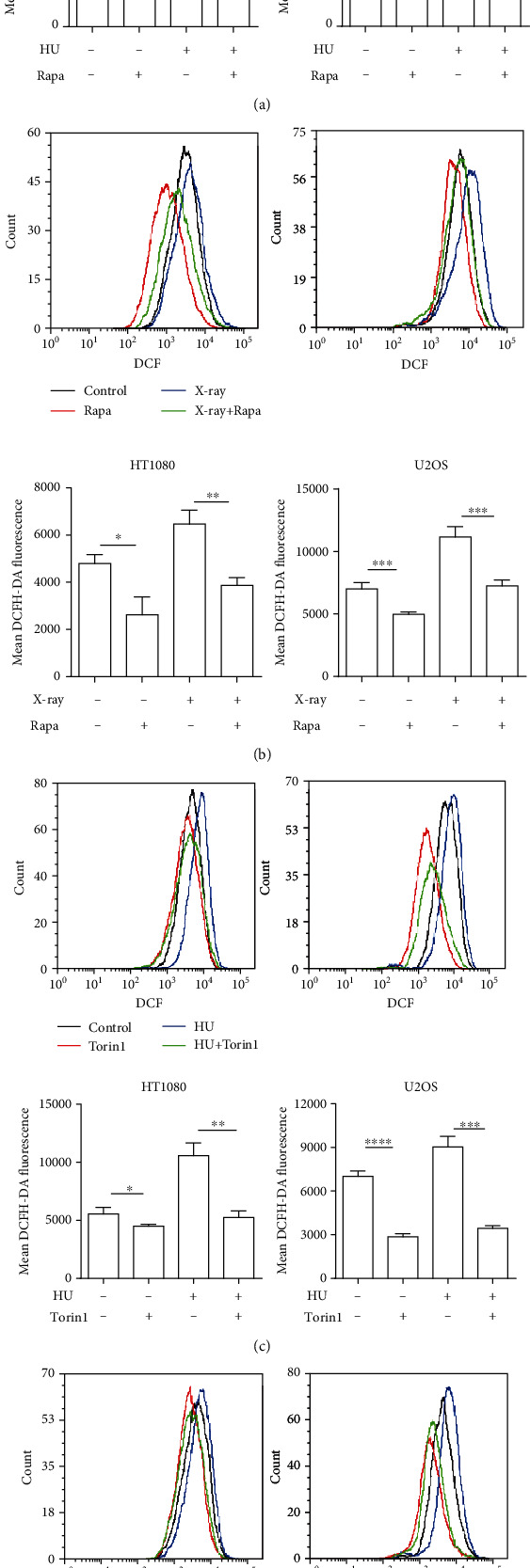
Autophagy reduces the ROS level of cancer cells. (a) Rapa reduces replication stress-induced ROS level of cancer cells. HT1080 cells and U2OS cells were treated with 20 nM Rapa or cotreated with 200 *μ*M HU for 24 h. Cellular ROS level was determined by flow cytometry. (b) Rapa reduces X-ray-induced ROS level of cancer cells. HT1080 cells and U2OS cells were pretreated with or without Rapa (20 nM) for 6 h and then were treated with 5 Gy X-ray. Cellular ROS level was tested at 24 h after X-ray treatment using flow cytometry. (c, d) Another autophagy activator Torin1 (50 nM) for verification in HT1080 cells and U2OS cells, which has the same treatment as (a) and (b). Each experiment was repeated three times. ^∗^*P* < 0.05, ^∗∗^*P* < 0.01, ^∗∗∗^*P* < 0.001, and ^∗∗∗∗^*P* < 0.0001. ROS: reactive oxygen species.

Redox homeostasis is critical for cell proliferation and survival. Excessive ROS are cytotoxic and can drive genomic instability [17, 19]. There are positive and negative reports on the regulation of redox by autophagy [15, 27, 28]. To determine whether the contribution of autophagy to genomic stability is related to redox homeostasis, we examined the ROS level in HT1080 cells and U2OS cells after rapamycin treatment. As shown in Figure 3(a), both the basal and HU-induced ROS levels were significantly reduced by rapamycin. Similarly, the elevation of ROS induced by X-ray was also attenuated by rapamycin (Figure 3(b)). Furthermore, Torin1 exhibited a similar effect on the basal and stress-induced ROS in HT1080 and U2OS cells (Figures 3(c) and 3(d)). These results suggest that autophagy functions to reduce the accumulation of ROS, a known driver of genomic instability.

### 3.4. The ROS and Micronucleation in Senescent Cells Are Attenuated by Rapamycin

**Figure 4:**
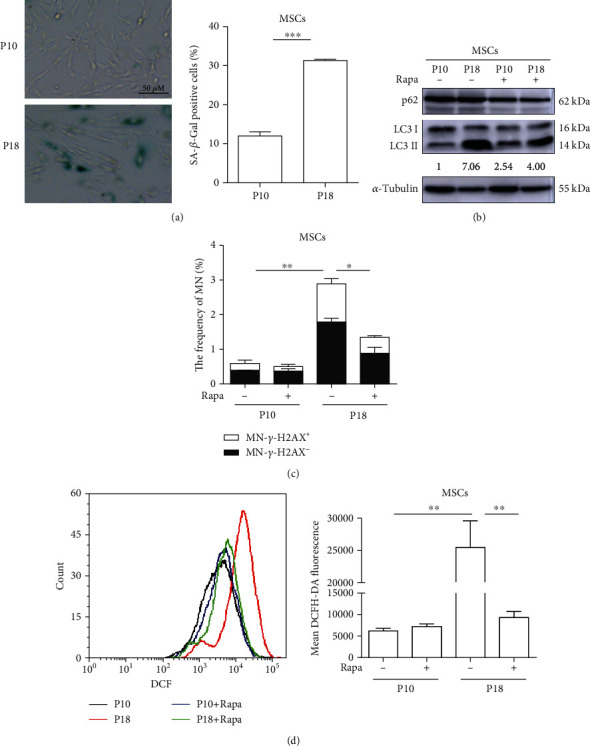
Induction of autophagy reduces ROS and micronuclei in senescent cells. (a) The percentage of senescent MSCs at passages 10 (P10) and 18 (P18). Cellular senescence was tested by SA-*β*-gal staining. (b) Rapa partially restores the autophagic flux in senescent cells. Young and senescent MSCs were treated with Rapa (20 nM) for 24 h for examination of autophagy efficiency (LC3 and p62) using Western blot analysis (the numbers below indicate ratios of LC3-II/LC3-I). (c) Rapa reduces micronuclei in senescent MSCs. Normal and senescent MSCs were treated with Rapa (20 nM) for 24 h; the frequencies of MN were examined at 48 h after Rapa was washed out. (d) Rapa reduces the ROS level of senescent cells. Normal and senescent MSCs were treated with Rapa (20 nM) for 24 h; cellular ROS level was measured by flow cytometry. Each experiment was repeated three times. ^∗^*P* < 0.05, ^∗∗^*P* < 0.01, and ^∗∗∗^*P* < 0.001. SA-*β*-gal: senescence-associated *β*-galactosidase.

Senescent cells generally show impaired autophagy flux [29]. In order to explore the role of the autophagy-redox link in the formation of MN in more contexts, we evaluated the effect of autophagy on micronucleation in senescent human umbilical cord mesenchymal stem cells (hUC-MSCs). As shown in Figure 4(a), the percentage of senescent cells was increased in late passage of MSCs when compared to early passage, as revealed through SA-*β*-gal staining. As shown in Figure 4(b), there was indeed more accumulation of LC3-II in senescent than in younger MSCs. Rapamycin restored the autophagic flux in senescent cells to a certain extent, as shown by the reduced accumulations of LC3-II and p62. As expected, senescent MSCs exhibited a significant elevation in micronuclei, but it could be attenuated by rapamycin (Figure 4(c)). Correspondingly, the elevated ROS in senescent cells were also reduced by rapamycin (Figure 4(d)). These results further demonstrate a possible role of autophagy-redox link in the maintenance of genomic stability.

### 3.5. Inhibition of Autophagy Leads to an Elevation in Micronuclei

**Figure 5:**
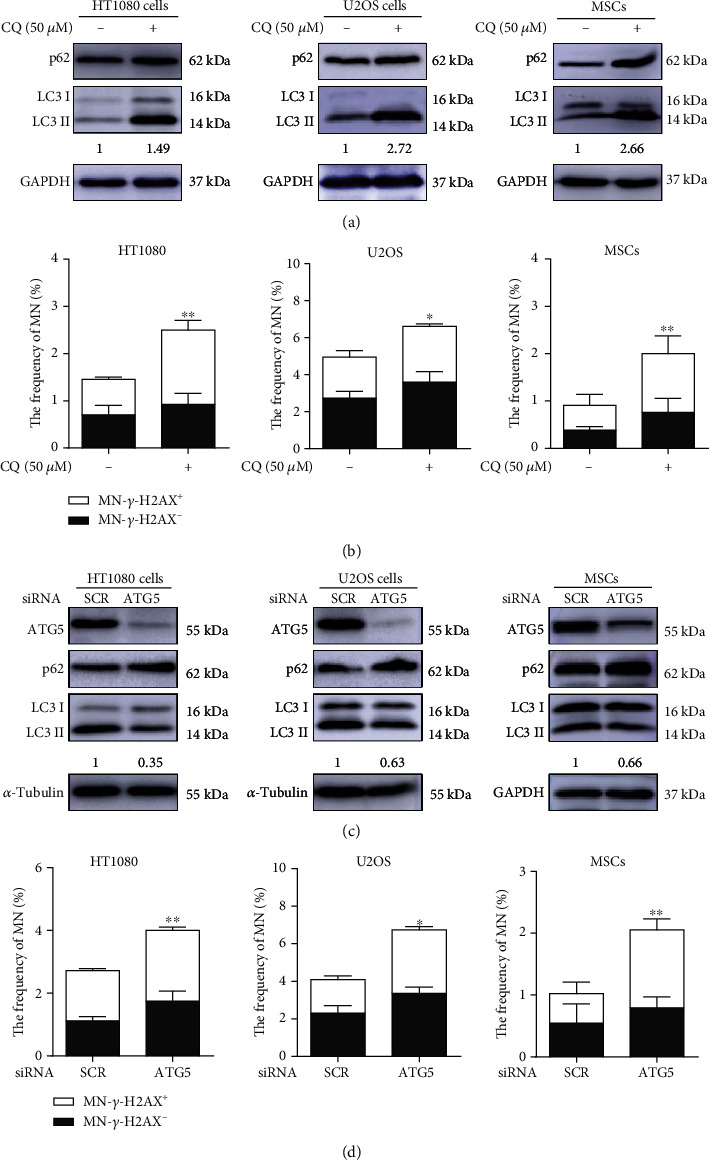
Blockade of autophagy increases micronuclei. (a) CQ treatment effectively blocked autophagic flux. HT1080, U2OS cells, and MSCs were treated with autophagy inhibitor CQ (50 *μ*M) for 24 h for examination of autophagy efficiency (LC3 and p62) by Western blot analysis (the numbers below indicate ratios of LC3-II/LC3-I). (b) CQ treatment increases micronucleation in HT1080 cells, U2OS cells, and MSCs. Cells were treated with CQ (50 *μ*M) for 24 h; the frequencies of MN were examined at 48 h after CQ was washed out. (c) ATG5 depletion inhibits the formation of early autophagosomes. HT1080, U2OS cells, and MSCs were treated with ATG5-siRNA or control-siRNA for 48 h for examination of interference efficiency and autophagy efficiency (LC3 and p62) by Western blot analysis (the numbers below indicate ratios of LC3-II/LC3-I). (d) Increased frequencies of micronuclei in ATG5 knockdown cells. Cells were treated with ATG5-siRNA or control-siRNA for 48 h for examination of micronuclei frequencies. Each experiment was repeated three times. ^∗^*P* < 0.05 and ^∗∗^*P* < 0.01. CQ: chloroquine; SCR: scrambled.

Chloroquine (CQ) is known to disrupt the function of lysosomes and thereby block autophagy [30]. We subjected cancer cells HT1080, U2OS, and MSCs to treatment with 50 *μ*M CQ and scored the MN. As shown in Figure 5(a), the treatment of CQ resulted in abnormal accumulations of LC3-II and the autophagy substrate p62 protein, indicating autophagy flux was efficiently blocked. As shown in Figure 5(b), there was a significant elevation in the frequencies of micronuclei upon CQ treatment. We further tested the effect of defective autophagy on micronucleation by depleting ATG5, which plays a key role in early autophagosome formation [31], with small interfering RNA. As shown in Figure 5(c), the depletion of ATG5 led to the reduction of LC3-II, but an increased accumulation of p62, indicative of a failure in forming autophagosome. As expected, the ATG5 depletion resulted in an increase in the frequencies of MN (Figure 5(d)).

### 3.6. Antioxidant Rescues Micronuclei Elevation Caused by Autophagy Inhibition

**Figure 6:**
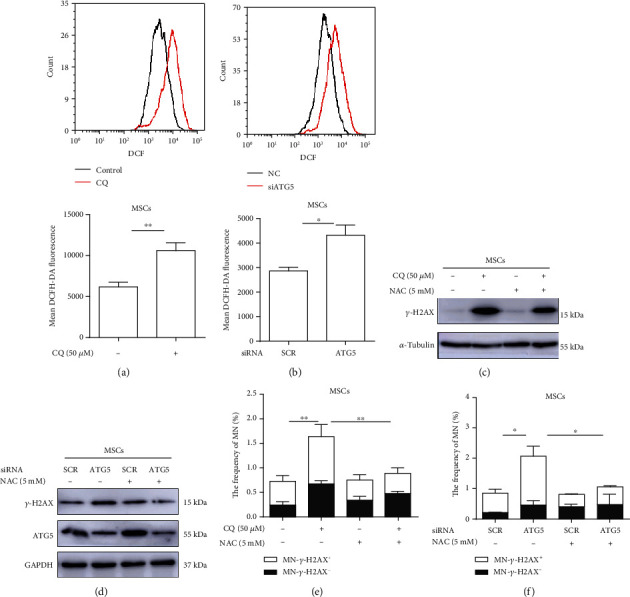
Oxidative stress mediates the elevation of MN caused by autophagy inhibition in MSCs. (a, b) Inhibition of autophagy increases ROS in MSCs. (a) MSCs were treated with CQ (50 *μ*M) for 24 h. (b) MSCs were transfected with ATG5 siRNA or control-siRNA, and cells were harvested for examination 48 h later. Cellular ROS level was determined by flow cytometry. (c, d) NAC reduces the accumulation of DNA damage caused by autophagy inhibition. (c) MSCs were pretreated with or without the antioxidant NAC (5 mM) for 2 h and then treated with CQ (50 *μ*M) for 24 h. (d) MSCs were transfected with ATG5-siRNA or control-siRNA and then treated with NAC (5 mM). The protein level of *γ*-H2AX was measured by Western blot analysis. (e, f) NAC attenuates MN elevation caused by autophagy inhibition. (e) MSCs were pretreated with or without the antioxidant NAC (5 mM) for 2 h and then treated with CQ (50 *μ*M) for 24 h; cells were processed for examination of the frequency of MN at 48 h after drugs were washed out. (f) MSCs were transfected with ATG5-siRNA or control-siRNA and then treated with NAC (5 mM) 24 h later; cells were processed for examination of the frequency of MN at 48 h after drug was washed out. Each experiment was repeated three times. ^∗^*P* < 0.05 and ^∗∗^*P* < 0.01. NAC: N-acetylcysteine.

We next tested whether the increased micronucleation by autophagy inhibition was accompanied by an elevation of ROS. As shown in Figures 6(a) and 6(b), autophagy inhibition led to a significant elevation in ROS in MSCs treated with CQ or ATG5 siRNA. However, such increase was not observed in the two cancer cell lines (Figures S2(a) and S2(b)), suggesting that whether or not inhibition of autophagy increases ROS may be cell-type specific. To determine whether the increased micronucleation in MSCs in which autophagy was inhibited was mediated by oxidative stress, we subjected MSCs to oxidant scavenger NAC. As shown in Figures 6(c) and 6(d), the level of *γ*-H2AX induced by autophagy inhibition was substantially reduced by NAC. Importantly, the MN induced by autophagy inhibition was abrogated by NAC (Figures 6(e) and 6(f)). These results suggest that oxidative stress mediates the increased micronucleation caused by autophagy inhibition in MSCs.

### 3.7. Autophagy Inhibits the Activation of CHK1

**Figure 7:**
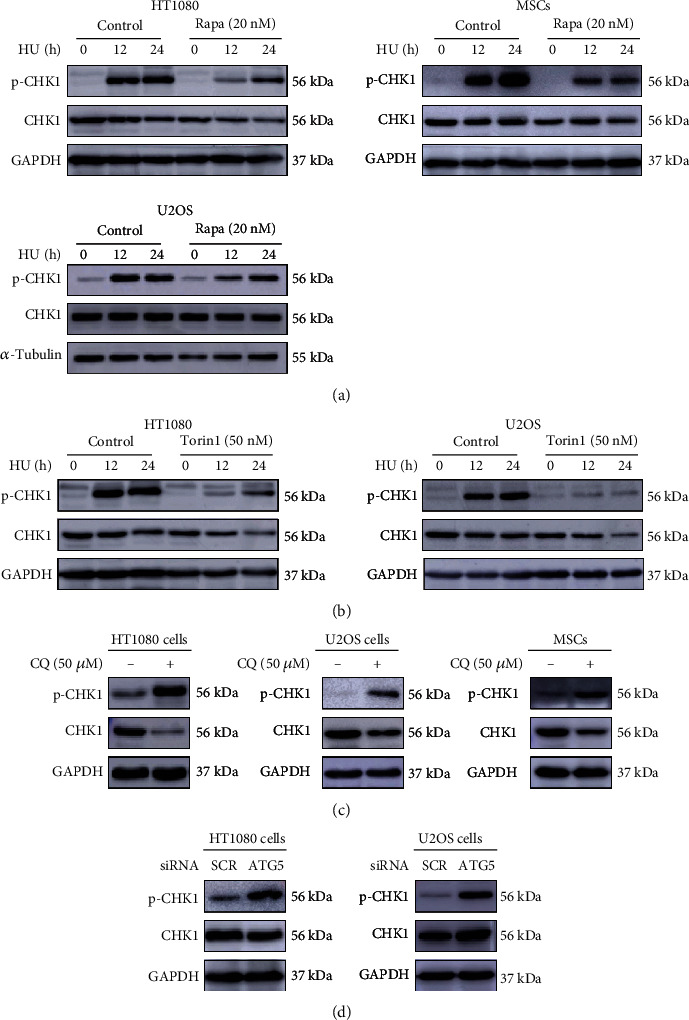
Autophagy inhibits the activation of CHK1. (a, b) Autophagy activators reduce p-CHK1 accumulation induced by HU. Phosphorylation of CHK1 at S345 and total CHK1 were determined by Western blot analysis in wild-type and Rapa-treated or Torin1-treated cells at the indicated times following exposure to 200 *μ*M HU. (c, d) Inhibition of autophagy leads to a significant upregulation of p-CHK1. (c) HT1080, U2OS cells, and MSCs were treated with CQ (50 *μ*M) for 24 h for measurement of the protein level of p-CHK1 and total CHK1 by Western blot analysis. (d) Western blot analysis of p-CHK1 and total CHK1 protein level in HT1080 and U2OS cells treated with ATG5 siRNA or control-siRNA for 48 h.

Checkpoint kinase 1 (CHK1) is a member of the serine/threonine protein kinase family and the core protein of cell cycle checkpoints in DNA damage responses. It was shown that the increased genomic instability in Atg7-deficient mouse embryonic fibroblasts was mainly due to the impairment of HR function caused by the decrease of p-CHK1 [12]. We wondered whether this mechanism also applies to the cells we studied here. As shown in Figures 7(a) and 7(b), autophagy activators rapamycin and Torin1 significantly reduced the level of p-CHK1 induced by HU. It should be noted that the decline in CHK1 activation was accompanied by a decrease in the frequency of micronuclei (Figures 2(c) and 2(d)). Furthermore, when autophagy was inhibited through CQ or ATG5 siRNA, there was an increased level of p-CHK1 (Figures 7(c) and 7(d)), which corresponded to an elevation in the frequency of micronuclei (Figures 5(b) and 5(d)). Together, these results suggest that the increased genomic instability caused by defective autophagy is probably independent of the reported CHK1-homologous recombination cascade.

## 4. Discussion

Autophagy plays a central role in regulating many important cellular functions. It has been reported that autophagy participates in degradation of RHOA and thereby maintains the appropriate level of active RHOA to ensure normal cell division [32]. Autophagy can also degrade retrotransposon RNA to reduce genomic instability [14]. Blockade of autophagy leads to the disorder of homologous recombination repair [12]. These processes may all be conducive to the maintenance of genome stability, but the relative contribution of these different processes to genome stability is not clear. Micronuclei have been widely used as an important indicator of genomic instability. Autophagy was shown to directly degrade micronuclei, as evidenced by the localization of GFP-LC3 or p62-SQSTM1 on micronuclei, but the percentage of “autophagic micronuclei” is relatively small (2-5%) [9, 33]. We here showed that autophagy activators can significantly reduce the spontaneous and genotoxic-induced micronuclei in cancer cells. They also attenuated the elevation of micronuclei in senescent MSCs. Therefore, our results suggest that autophagy can reduce the frequency of micronuclei indirectly, via alleviating oxidative stress that drives the formation of micronuclei.

Because oxidative stress is known to be a driver of genomic instability, the reduction of ROS in cancer cells and senescent MSCs by augmented autophagy is expected to contribute to the maintenance of genomic stability. It appears that whether the increased micronucleation caused by inhibition of autophagy is mediated by increased oxidative stress is context-dependent. In the two cancer cell lines we examined, the induction of MN by inhibition of autophagy is not accompanied by ROS elevation. However, inhibition of autophagy did result in the elevation of ROS in MSCs, and the induction of micronuclei by the impaired autophagy could be effectively blocked by antioxidant. It should be noted that the redox regulation by autophagy may operate by complex and diverse mechanisms. Autophagy defects lead to the accumulation of ROS, indicating that autophagy negatively regulates ROS [21, 22]. However, upon RAS overexpression, autophagy degrades Lamin B1 and then drives senescence, presumably via increased ROS [34]. The results we presented here indicated that the disruption of redox homeostasis is an important mediator of genomic instability caused by autophagy defect under certain conditions.

It was proposed that inhibition of autophagy caused by overexpression of miR-20a or interference with its target genes can drive the accumulation of ROS and DNA damage, which likely contributes to tumor initiation [35], but the causal relationship between the ROS elevation and the accumulation of DNA damage as a consequence of defective autophagy was unclear. Importantly, our results show that the increased micronucleation in MSCs in response to autophagy inhibition was efficiently blocked by antioxidant NAC, which provides definitive evidence that increased ROS can drive genomic instability when autophagy is impaired. Our study provides more insight into the mechanism by which autophagy contributes to genomic stability.

Impaired autophagy was shown to decrease CHK1 in response to stress and thereby greatly diminish the ability to repair DNA double-strand breaks by homologous recombination (HR) [12]. In this study, we observed that autophagy activation instead resulted in a decrease in the protein level of p-CHK1, which in turn was accompanied by a decrease in frequency of micronuclei. Blockade of autophagy, on the contrary, leads to an increased accumulation p-CHK1 and increased frequency of micronuclei. Because CHK1 is activated in response to oxidative stress and oxidative DNA damage [19], the decrease or increase in the level of activated CHK1 when autophagy is augmented or inhibited may simply reflect the degree of oxidative DNA damage the cells incur. Thus, the increased micronucleation associated with autophagy inhibition is unlikely to be caused by a failure in the CHK1-homologous recombination cascade. The complexity in the relationship between DNA damage, CHK1 dynamics, and autophagy is further illustrated by the report that the phosphorylation of CHK1 caused by DNA damage can eventually lead to the degradation of the total amount of CHK1 [36]. Interestingly, prolonged persistence of CHK1 in the nucleus, as when the ability of chaperone-mediated autophagy (CMA) to selectively degrade CHK1 was impeded, even drives the accumulation of DNA damage and the instability of MRN complex [37]. Therefore, the regulatory effect of autophagy on CHK1 and its role in DNA damage response may vary with experimental settings and/or cell lines, which needs to be further clarified. It should be pointed that the changes in the total CHK1 are generally less pronounced than those in the p-CHK1 under the different experimental conditions, as shown in Figure 7.

To sum up, this study explores the role of autophagy-regulated redox homeostasis in the maintenance of genomic stability using micronuclei as an indicator. The results show that redox homeostasis sustained by autophagy significantly contributes to the maintenance of genome stability.

## Data Availability

The data used to support the findings of this study are available from the corresponding author upon request.

## Supplementary Materials

Supplementary Materials Figure S1: autophagy reduces X-ray-induced micronuclei. (a, b) HT1080 cells and U2OS cells were pretreated with or without autophagy activator Rapa (20 nM) for 6 h and then were treated with 5 Gy X-ray. Cells were fixed for measurement of the frequency of MN at 48 h after Rapa was washed out. ^∗^*P* < 0.05 and ^∗∗^*P* < 0.01. Figure S2: inhibition of autophagy in cancer cells without elevated ROS. (a) HT1080 and U2OS cells were treated with CQ (50 *μ*M) for 24 h for examination of ROS using flow cytometry. (b) HT1080 cells and U2OS cells were transfected with ATG5 siRNA or Control-siRNA, and cells were harvested for test 48 h late.
