# Supplementary material for: Autophagy Contributes to the Maintenance of Genomic Integrity by Reducing Oxidative Stress
Source: Oxid Med Cell Longev. 2020 Aug 25;2020:2015920. doi: 10.1155/2020/2015920 (PMC7471819; doi:10.1155/2020/2015920)
Supplement: Supplementary Materials — Figure S1: autophagy reduces X-ray-induced micronuclei. (a, b) HT1080 cells and U2OS cells were pretreated with or without autophagy activator Rapa (20 nM) for 6 h and then were treated with 5 Gy X-ray. Cells were fixed for measurement of the frequency of MN at 48 h after Rapa was washed out. ∗P < 0.05 and ∗∗P < 0.01. Figure S2: inhibition of autophagy in cancer cells without elevated ROS. (a) HT1080 and U2OS cells were treated with CQ (50 μM) for 24 h for examination of ROS using flow cytometry. (b) HT1080 cells and U2OS cells were transfected with ATG5 siRNA or Control-siRNA, and cells were harvested for test 48 h late. [file 2015920.f1.pdf]

Supplementary Materials:

FIGURE S1. Autophagy reduces X-Ray induced micronuclei. (a-b) HT1080 cells and U2OS cells were pretreated with or without autophagy activator Rapa (20 nM) for 6 h, and then were treated with 5 Gy X-ray. Cells were fixed for measurement of the frequency of MN at 48 h after Rapa was washed out. *P < 0.05, **P < 0.01.

FIGURE S2. Inhibition of autophagy in cancer cells without elevated ROS. (a) HT1080 and U2OS cells were treated with CQ (50 μM) for 24 h for examination of ROS using Flow cytometry. (b) HT1080 cells and U2OS cells were transfected with ATG5 siRNA or Control-siRNA, and cells were harvested for test 48 h later.

Figure S1

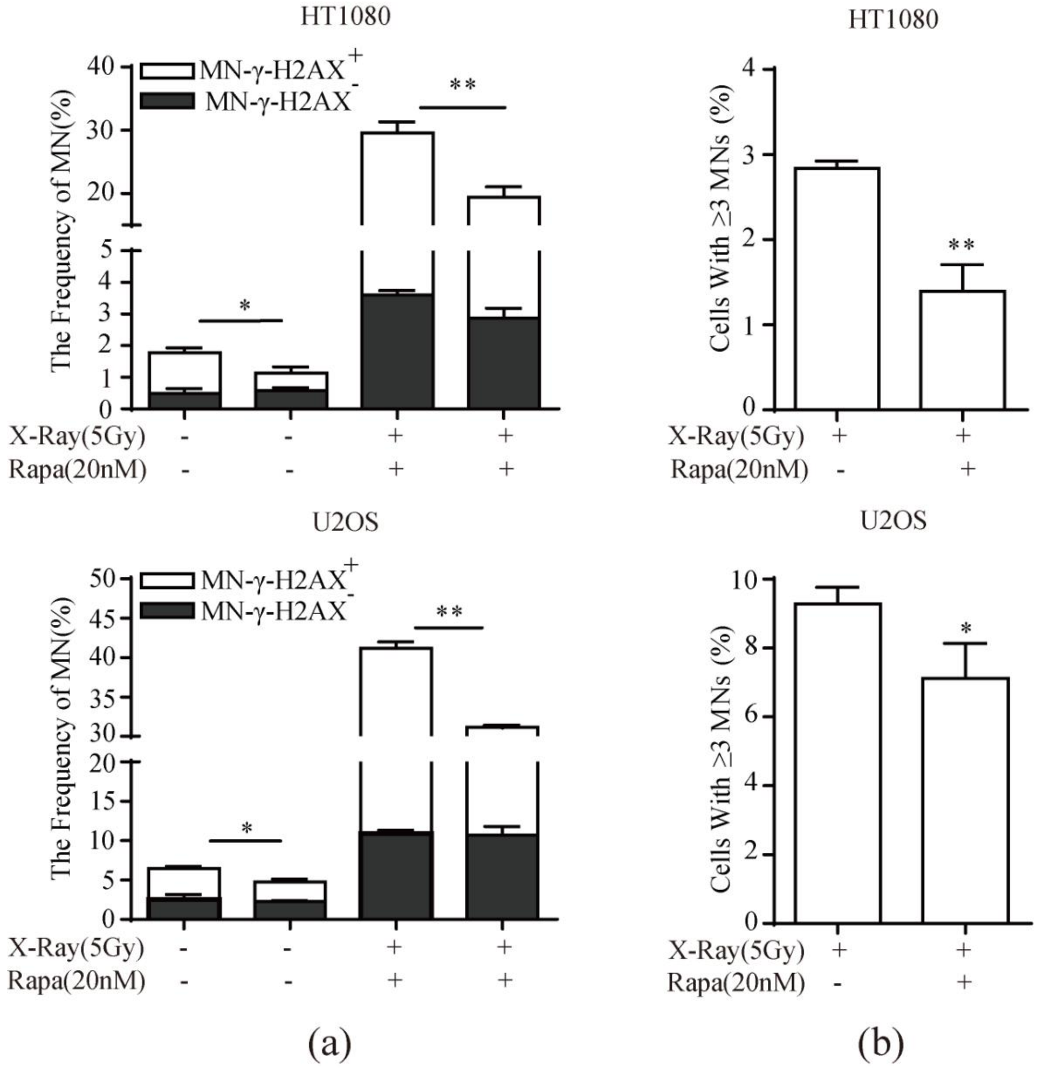
HT1080
MN-γ-H2AX+
MN-γ-H2AX-
The Frequency of MN(%)
Cells With ≥3 MNs (%)
X-Ray(5Gy)
Rapa(20nM)
U2OS
(a)
(b)

Figure S2

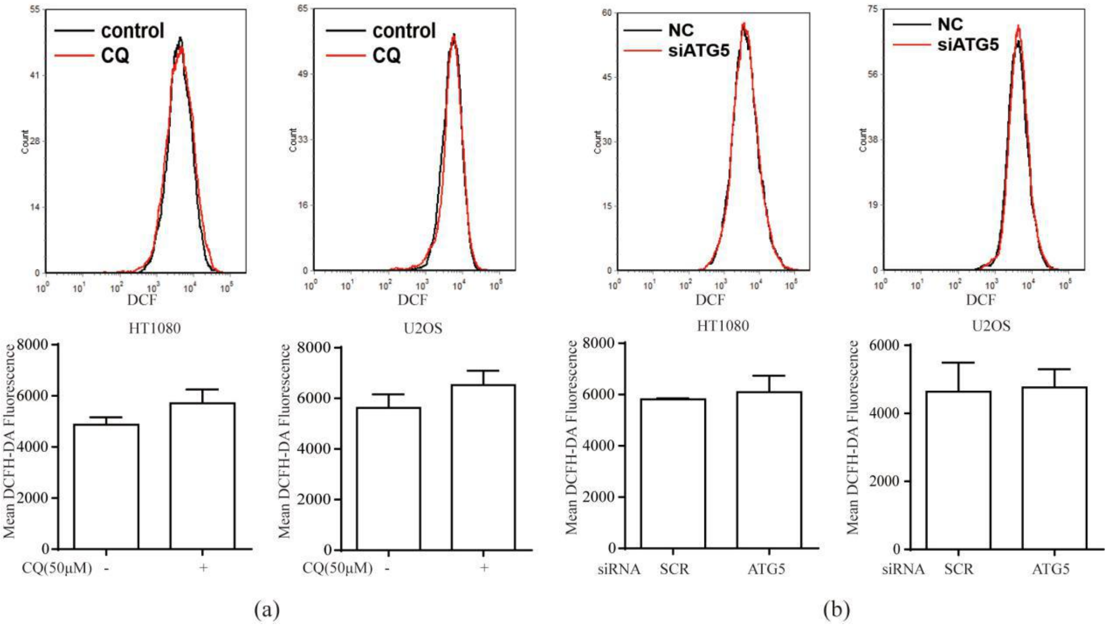
control
CQ
control
CQ
NC
siATG5
NC
siATG5
Count
DCF
HT1080
U2OS
HT1080
U2OS
Mean DCFH-DA Fluorescence
CQ(50μM) - +
CQ(50μM) - +
siRNA SCR ATG5
siRNA SCR ATG5
(a)
(b)
